# Blockade of NLRP3 alleviates systemic lupus erythematosus-induced osteoporosis through dual modulation of osteogenesis and osteoclastogenesis

**DOI:** 10.1016/j.bonr.2026.101942

**Published:** 2026-08-22

**Authors:** Feifu Deng, Yuting Liao, Weixiong Guo, Xiangxin Zhong, Xiang Gao, Ahmad, Yanzhi Liu, Jinsong Wei

**Affiliations:** a Orthopedics department, The Affiliated Hospital of Guangdong Medical University, Zhanjiang, 524001, People's Republic of China; b Key Laboratory of Traditional Chinese Medicine for the Prevention and Treatment of Infectious Diseases, Institute of Clinical Medicine, Zhanjiang Central Hospital, Guangdong Medical University (Central People's Hospital of Zhanjiang), Zhanjiang, 524045, People's Republic of China; c Marine Medical Research Institute of Zhanjiang, School of Ocean and Tropical Medicine, Guangdong Medical University, Zhanjiang, 524023, People's Republic of China; d School of Pharmacy, Guangdong Medical University, Zhanjiang, 524023, People's Republic of China; e Stem Cell Research and Cellular Therapy Center, The Affiliated Hospital of Guangdong Medical University, Zhanjiang, 524001, People's Republic of China

**Keywords:** Lupus erythematosus, Osteoporosis, NLRP3, MCC950, Oridonin, MRL*/lpr* mice

## Abstract

Systemic lupus erythematosus (SLE) is a chronic autoimmune disease frequently associated with accelerated bone loss and osteoporosis. Emerging evidence implicates the NLRP3 inflammasome as a pivotal mediator of inflammation-driven bone remodeling dysregulation in SLE. This study investigates the therapeutic potential of targeting NLRP3 inhibition for SLE treatment using both a synthetic NLRP3 inhibitor (MCC950) and a natural NLRP3 inhibitor—oridonin, for its potential clinical translation.

Using a combination of *in vitro* and *in vivo* approaches, we demonstrate that NLRP3 activation by lipopolysaccharide (LPS) promotes osteoclast differentiation and inhibits osteogenic differentiation through distinct molecular mechanisms. In RAW264.7 macrophages, LPS-induced NLRP3 activation upregulates autophagy-related genes (LC3B, p62) and osteoclastogenesis- related genes (TRAF6, c-Jun, NFATc1), which are effectively suppressed by MCC950 intervention. In mesenchymal stem cells, LPS exposure impairs osteogenic differentiation by reducing Runx2 and OCN gene expression while increasing pyroptosis-related genes (GSDMD, Caspase-11), these effects that are significantly ameliorated by oridonin intervention.

In an SLE murine model (MRL/lpr mice), 12 weeks of NLRP3 inhibition with either MCC950 or oridonin both restore bone microstructure, enhance trabecular bone density, improve biomechanical properties, and normalize serum biomarkers of bone turnover.

This study provides compelling evidence that targeting NLRP3 signaling represents a promising therapeutic strategy for SLE-associated osteoporosis. The osteogenic effect of oridonin, a naturally occurring compound with established clinical safety, highlights its potential as a promising agent for treating bone loss in SLE patients. These findings underscore the importance of inflammasome targeting in managing SLE-induced bone remodeling disorders.

## Highlights

• NLRP3 drives aberrant osteogenic and osteoclastic lineage commitment in lupus-induced bone deterioration;
• NLRP3 Inflammasome signaling directs MSC lineage allocation, revealing mechanisms of inflammatory bone biology;
• Repurposing oridonin offers a targeted clinical strategy to correct lupus-induced maladaptive bone remodeling;

## 1 Introduction

Osteoporosis (OP) is a systemic skeletal disorder characterized by reduced bone mass, deteriorated bone microarchitecture, and increased bone fragility (Song et al., 2022). Secondary osteoporosis is commonly observed in patients with systemic lupus erythematosus (SLE), primarily due to elevated levels of pro-inflammatory cytokines associated with SLE (Umare et al., 2014; Adami et al., 2019; Nepal and Gazeley, 2023). Chronic systemic inflammation disrupts the coupling between osteoclasts and osteoblasts, leading to bone metabolic imbalance and bone loss (Bultink, 2018). Current management strategies for SLE-induced osteoporosis rely predominantly on standard osteoporosis agents (*e.g.*, bisphosphonates, teriparatide, and denosumab). While these agents provide modest therapeutic benefits, they do not target the underlying inflammatory milieu that accelerates bone resorption in SLE, highlighting an urgent need for pathway-specific interventions to address SLE-associated osteopenia. Additionally, traditional treatments are associated with potential complications: bisphosphonates carry a risk of osteonecrosis of the jaw and atypical femoral fractures (Shane et al., 2014; Vermeer et al., 2016), teriparatide is expensive and also carries a risk of osteosarcoma, and denosumab may cause hypocalcemia (Bridgeman and Pathak, 2011). Given the unique inflammatory microenvironment of SLE, there is an urgent need to explore novel, targeted therapies for alleviating SLE-associated osteoporosis.

The NOD-like receptor family pyrin domain-containing 3 (NLRP3) inflammasome is a multiprotein complex that regulates the caspase-1-dependent maturation and secretion of pro-inflammatory cytokines interleukin-1β (IL-1β) and IL-18, thereby driving inflammatory responses (Yang et al., 2019). IL-1β interacts with receptors on macrophages to promote RANKL production, which subsequently binds to RANK on osteoclast precursors, leading to osteoclast activation and differentiation, and ultimately increasing bone resorption (Ruscitti et al., 2015; Yao et al., 2021). The NLRP3 inflammasome indirectly suppresses osteoblast differentiation by inhibiting SIRT1, which normally promotes osteoblast differentiation by repressing adipocyte differentiation in mesenchymal stem cells (MSCs) (Wang et al., 2017). Thus, the NLRP3 inflammasome plays a pivotal role in both bone resorption and bone formation, making it a potential therapeutic target for the treatment of osteoporosis.

The NLRP3 inflammasome is also implicated in the pathogenesis of SLE. Elevated NLRP3 expression has been observed in macrophages, peripheral monocytes, podocytes, and tubular cells of SLE patients (Li et al., 2020). In SLE murine models, NLRP3 gene mutation exacerbates renal damage, and NLRP3 inhibition in MRL/*lpr* mice reduces the progression of lupus nephritis (Lu et al., 2017; Guo et al., 2019). These findings suggest that targeting NLRP3 inflammasome activation may offer dual therapeutic benefits by simultaneously alleviating inflammation and SLE-associated osteoporosis.

MCC950, a specific small molecule inhibitor of NLRP3 inflammasome activation, has shown therapeutic potential in various conditions, including autoimmune diseases, cardiovascular disorders, and metabolic diseases, although its precise mechanism of action remains under investigation (Li et al., 2022a). Oridonin, a diterpenoid compound derived from the traditional Chinese herb Rabdosia rubescens, exhibits diverse pharmacological activities, including anti-inflammatory, anticancer, cardioprotective, and renoprotective effects (Li et al., 2021). Accumulating evidence establishes oridonin as a naturally occurring NLRP3 inhibitor that suppresses inflammasome activation by disrupting the critical NLRP3–NEK7 interaction (He et al., 2018). Beyond its traditional clinical application in China for treating pharyngolaryngeal inflammation, oridonin's potent regulatory control over the NLRP3 axis presents a compelling case for drug repurposing. Unlocking this mechanism transforms this traditional compound into a strategically viable candidate for combating systemic inflammation.

In this study, we aim to investigate the potential therapeutic effects of target blocking the NLRP3 inflammasome in the treatment of SLE and SLE-induced osteoporosis, with a particular focus on oridonin as a promising “old drug for new use” candidate. This research seeks to uncover a novel therapeutic strategy for the prevention and management of SLE-associated osteoporosis.

## 2 Materials and methods

### 2.1 Cell culture

C3H10 cells and RAW 264.7 macrophages were purchased from Wuhan Pricella Biotechnology (Wuhan, China). C3H10 cells were cultured in specialized MSC medium (DMEM [PM150210] supplemented with 10% FBS [164210] and 1% penicillin-streptomycin [PB180120]; Wuhan Pricella, China) at 37 °C in a humidified atmosphere containing 5% CO_2_. RAW 264.7 macrophages were maintained in MEM supplemented with non-essential amino acids (NEAA) [PM150410], 10% FBS [164210–50], and 1% penicillin-streptomycin [PB180120] (Wuhan Pricella, China) under identical conditions. The culture medium for C3H10 cells was replaced every two days. Upon reaching 90% confluence, C3H10 cells were detached using 0.25% trypsin (Gibco) and subcultured at a 1:3 ratio. RAW 264.7 macrophages were passaged every two days at a 1:6 ratio.

### 2.2 Cell viability analysis

Cell viability was assessed using the CCK-8 assay (Beyotime). For RAW 264.7 macrophages, the effects of lipopolysaccharide (LPS, Sigma) and MCC950 (MedChemExpress) were evaluated. C3H10 cells were treated with LPS alone, and their viability was measured using the same method. In brief, RAW 264.7 macrophages were plated into 96-well plates at 5 × 10^3^ cells per well and allowed to settle for 24 h. Subsequently, the cells were exposed to varying concentrations of LPS (0.001-20 μg/mL) and MCC950 (0.01-200 μM) for 24, 48, or 72 h. In each experimental group, six replicate wells receiving vehicle only (0 μM) served as controls. For C3H10 cells, 2 × 10^3^ cells per well were incubated with a range of LPS concentrations over the same three time points. Following treatment, 10 μL of CCK-8 solution was added to every well, and the plates were kept at 37 °C for an additional 2 h. Absorbance at 450 nm was then recorded using a microplate reader (Bio-Rad Laboratories, Hercules, CA, USA).

### 2.3 Real-time quantitative PCR (qRT-PCR)

RAW264.7 cells (6 × 10^5^ cells/well) were cultured in six-well plates and treated with lipopolysaccharide (LPS) at a concentration of 0.1 μg/mL, receptor activator of nuclear factor kappa-B ligand (RANKL) at 100 ng/mL (PeproTech), and compound MCC950 at 10 μM for 48 h. Similarly, the C3H10 cells (2 × 10^5^ cells/well) were seeded and subjected to treatment with LPS at 10 μg/mL and compound oridonin at 1 μM for either 24 h or 7 days. Following treatment, cells were harvested, and total cellular RNA was extracted using a small volume total RNA preparation kit (Corning, USA, AP-MN-MS-RNA-250). cDNA was synthesized using the PrimeScript™ RT Master Mix reagent kit (Takara). Real-time PCR was conducted using TB Green Premix EX Taq II (Takara), and data analysis was performed using the 2^−ΔΔCT^ method. Each experiment was conducted in triplicate.

### 2.4 Western blot

After treating RAW 264.7 macrophages with 0.1 μg/mL LPS, 100 ng/mL RANKL, and 10 μM MCC950 for 72 h, and C3H10 cells with 10 μg/mL LPS and 1 μM ORI for 7 days, western blot analysis was performed on both cell types. Total protein was extracted using RIPA lysis buffer (Beyotime, China), and protein concentrations were quantified using a BCA assay kit (Beyotime, China). The samples were then separated by SDS-PAGE and transferred onto PVDF membranes. Following a 1-h incubation with blocking buffer (Beyotime, China), the membranes were incubated overnight at 4 °C on a shaker with specific primary antibodies: GAPDH (#52902, SAB, USA),), NLRP3 (#15101, CST, USA), C-JUN (#9165, CST, USA), p62 (#23214, CST, USA), LC3 A/B (#4108, CST, USA), IL-6 (ab22938, Abcam, UK), RUNX2 (ab23981, Abcam, UK), Osterix (ab94744, Abcam, UK), and OCN (ab93876, Abcam, UK). After washing, a diluted secondary antibody (Proteintech, China) was applied and incubated at room temperature for 2 h. Subsequently, ECL hypersensitive luminescence solution (Beyotime, China) was used to visualize the protein bands, which were captured using a gel imager. ImageJ software was utilized to analyze the gray values of the protein bands and calculate relative expression levels. Each western blot was conducted in triplicate.

### 2.5 TRAP staining

RAW264.7 cells were plated at a density of 5 × 10^3 cells per well in 24-well plates and stimulated with RANKL at a concentration of 100 ng/mL. After 6 days, the cells were washed with PBS, fixed with 4% paraformaldehyde (PFA) on ice for 20 min, and then stained using the TRAP Staining Kit (Takara, MK300) following the manufacturer's instructions. Cells exhibiting more than three nuclei and pronounced red cytoplasmic staining were classified as mature osteoclasts. The total stained area per well was analyzed using ImageJ software.

### 2.6 Animal study protocol

Female C57BL/6 mice (*n* = 20) and MRL/MpJ-Faslpr (MRL*/lpr*, *n* = 40) at 6–8 weeks of age were obtained from Nanjing Junke Bioengineering Co., Ltd. (Certificate No. SCXK (苏) 2020–0009). The mice were acclimatized for 7 days in ventilated cages under controlled conditions (temperature 22 ± 2 °C, 12-h light/dark cycle) with *ad libitum* access to food and water. All experimental procedures were performed in accordance with the Guide for the Care and Use of Laboratory Animals (8th edition, NIH) and were approved by the Animal Ethics Committee of Guangdong Medical University (No. AHGDMU-LAC-B-202208-0029 and No. AHGDMU-LAC-B-202208-0016).

The treatment protocols for the MCC950 group are as follows: Control (C57BL/6 mice + normal saline, *n* = 10), SLE model (MRL*/lpr* mice + normal saline, *n* = 10), and SLE + MCC950(MRL*/lpr* mice +10 mg/kg MCC950, *n* = 10). The treatment protocols for the Oridonin group consist of: Control (C57BL/6 mice + normal saline, *n* = 10), SLE model (MRL*/lpr* mice + normal saline, *n* = 10), and SLE + ORI (MRL*/lpr* mice +10 mg/kg ORI, *n* = 10). All therapeutic agents were administered *via* intraperitoneal injection (i.p.) three times a week for 12 consecutive weeks.

### 2.7 Collection and processing of samples

After 12 weeks of treatment, all mice were euthanized. Blood samples were collected *via* cardiac puncture and centrifuged at 3000 rpm for 30 min to obtain serum. The femurs were harvested, fixed in 4% paraformaldehyde, and prepared for micro-CT imaging and biomechanical analysis. Serum levels of β-C-terminal telopeptide of type I collagen (β-CTX) and N-terminal propeptide of type I procollagen (PINP) were measured using commercial ELISA kits (β-CTX: Nanjing Jiancheng Bioengineering Institute, *E*-EL-M3075; PINP: Nanjing Jiancheng, E-EL-M3069) following the manufacturer's instructions.

### 2.8 Micro CT analysis

After the left femur of the mice was fixed in a 4% solution of paraformaldehyde for 30 h, Micro-CT analysis was performed using a Viva CT80 scanner (Scanco, Switzerland) with the following parameters: 2048 × 2048 acquisition matrix, 70 kVp energy, 114 μA intensity, 8 W power, and an integration time of 200 ms. The region of interest (ROI) for cancellous bone was defined as the bone tissue located 1.0 mm from the distal femoral growth plate, which was scanned along the long axis of the femur, allowing for three-dimensional reconstruction of the images. Quantitative analysis of the acquired images was conducted using the analysis software provided with the system, focusing on parameters such as bone volume fraction (BV/TV), trabecular thickness (Tb. Th), trabecular number (Tb. N), trabecular separation (Tb. Sp), and the structural model index (SMI).

### 2.9 Measurement of the biomechanical properties

The biomechanical properties of the right femurs in mice were evaluated using a three-point bending test. Isolated femurs were placed on a support with a 10 mm span, with the midshaft serving as the loading point. Vertical compression was applied at a constant rate of 1 mm/min until fracture occurred. The load (F) *versus* deflection (d) curves were recorded, and key mechanical parameters—including maximum load, ultimate load, elastic load, and stiffness—were subsequently calculated.

### 2.10 Histopathology of the kidneys in mice

Renal specimens were harvested from mice, fixed in 4% paraformaldehyde for 24 h, embedded in paraffin, and sectioned at 4 μm thickness. Sections were then stained with hematoxylin and eosin (H&E). Histopathological changes were evaluated under a light microscope at 200× magnification in a blinded manner.

### 2.11 2.10 statistical analysis

The data were analyzed using SPSS version 29.0 and are presented as mean ± standard deviation (SD). One-way ANOVA was employed to compare differences among multiple groups. When homogeneity of variance was confirmed, Fisher's Least Significant Difference (LSD) method was applied. In cases of variance heterogeneity, Tamhane's T2 procedure was utilized as an alternative. A *p*-value of <0.05 was considered indicative of a statistically significant difference.

## 3 Results

### 3.1 High-dose MCC950 induces cytotoxicity in RAW264.7 cells

**Fig. 1:**
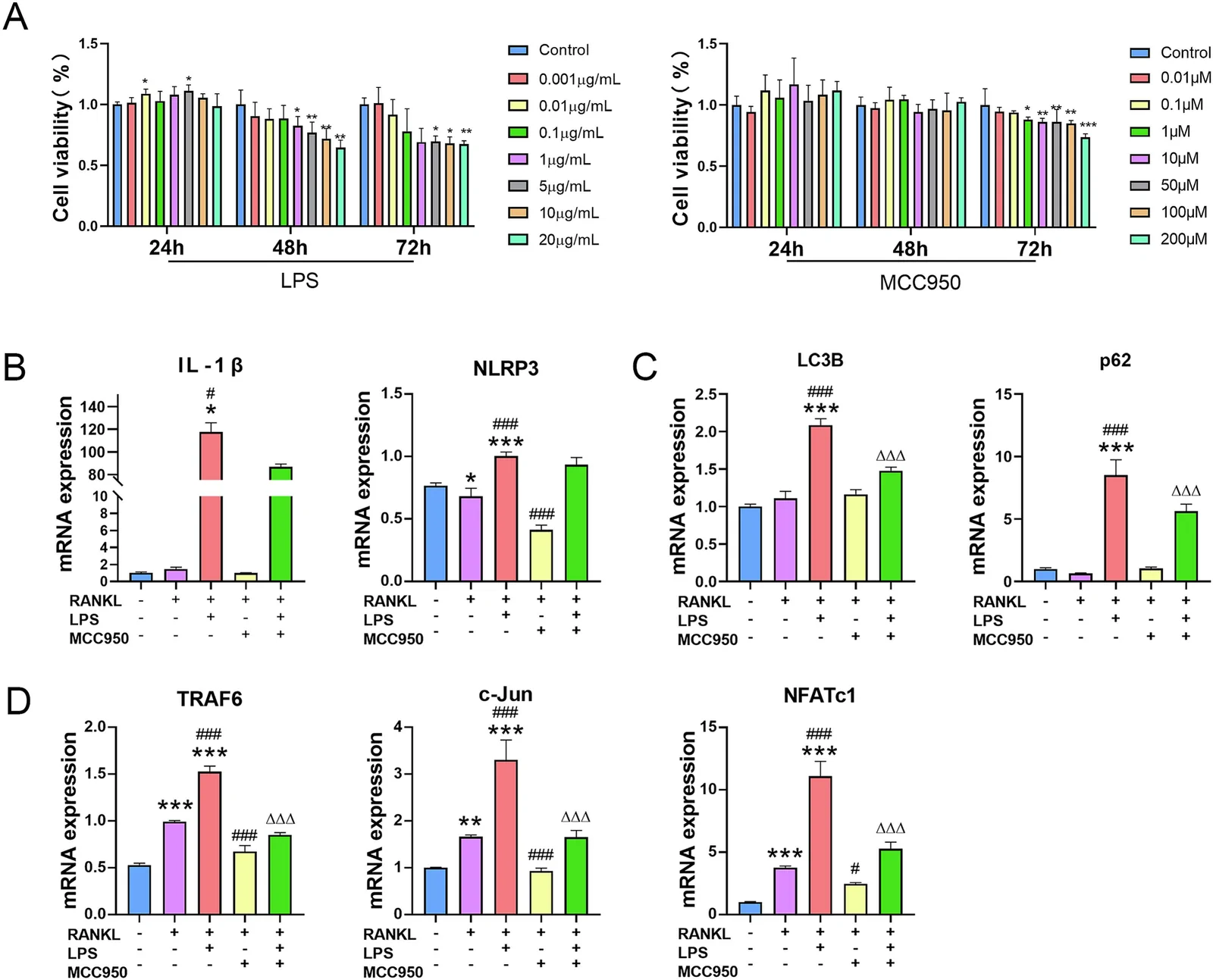
Effects of LPS, RANKL, and MCC950 treatments on RAW264.7 cells. (**A**) Cell viability measured by CCK-8 assay after LPS and MCC950 treatment. (**B, C, D**) mRNA expression levels of inflammation-related genes *IL-1β, NLRP3,* of autophagy-related genes *LC3B, p62*, osteoclast-specific genes *TRAF6, c-Jun, NFATc1* in RAW264.7 cells treated with LPS, RANKL, and MCC950 for 48 h.*P < 0.05, **P < 0.01, ***P < 0.001 *vs* Control;#*P* < 0.05, ##*P* < 0.01, ###*P* < 0.001 *vs* RANKL; Δ*P* < 0.05, ΔΔ*P* < 0.01, ΔΔΔ*P* < 0.001 *vs* RANKL + LPS (*n* = 3).

To establish baseline biocompatibility, we first quantified the impact of LPS and MCC950 on RAW264.7 cell viability *via* a Cell Counting Kit-8 (CCK-8) assay. As shown in Fig. 1A,Treatment with 10 μM MCC950 exerted no observable cytotoxic effects on RAW264.7 macrophages at 24 and 48 h; however, a time-dependent reduction in cell viability was detected following 72 h of continuous exposure (Fig. 1A). Guided by prior report from Cai et al. (Cai et al., 2024). and our baseline viability data, we selected 10 μM as the optimal working concentration of MCC950 to attenuate LPS-induced inflammatory activation in RAW264.7 cells.

### 3.2 MCC950 attenuates LPS-induced inflammation and autophagy in RAW264.7 cells

**Fig. 2:**
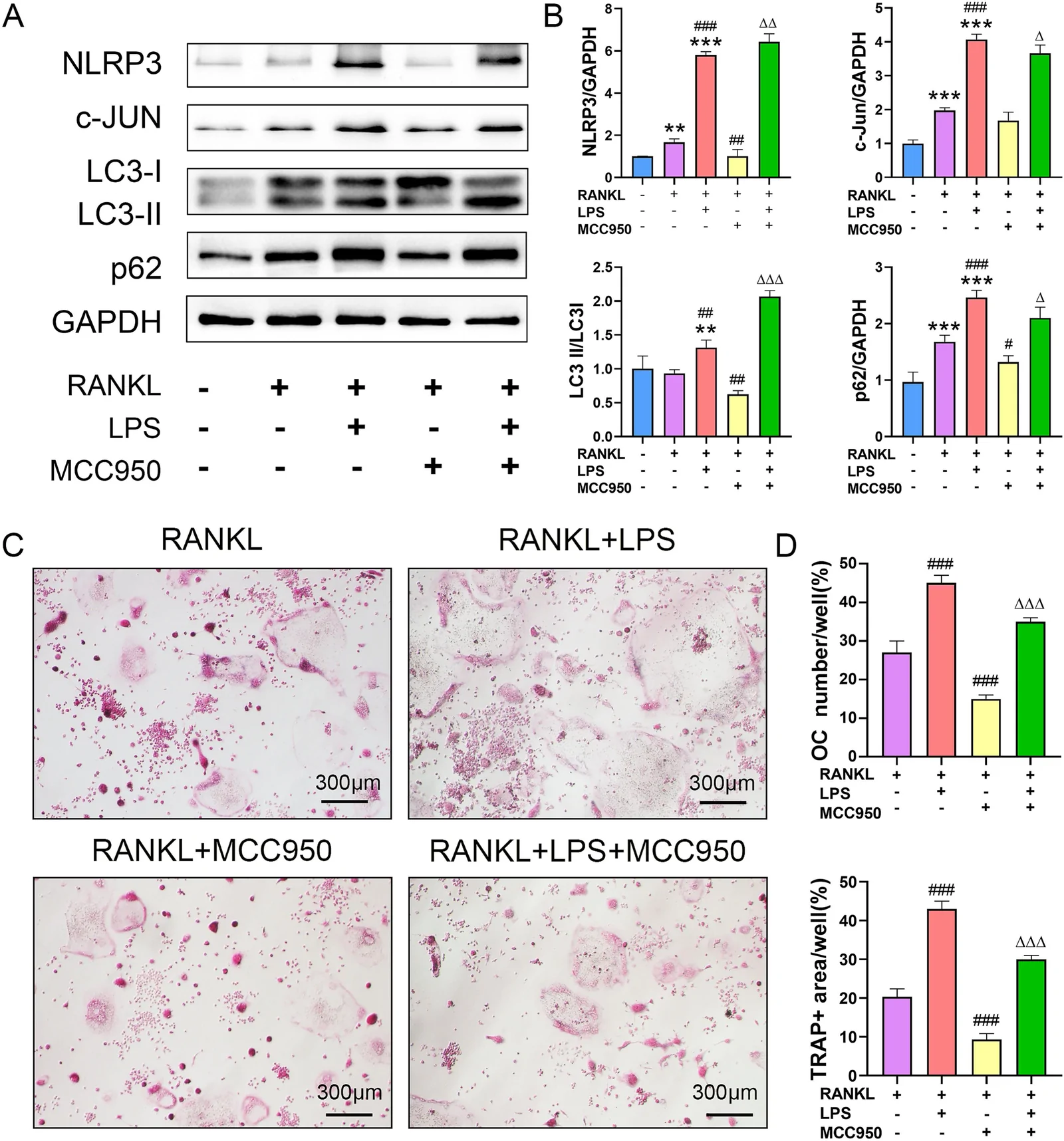
MCC950 inhibits osteoclast differentiation of RAW264.7 cells. (**A**) Protein expression of NLRP3, c-Jun, and LC3B in RAW264.7 cells treated with LPS, RANKL, and MCC950 for 72 h. (**B**) Quantitative analysis of Western blot results. (**C**, **D**) Osteoclastogenesis identified by TRAP staining. *P < 0.05, **P < 0.01, ***P < 0.001 *vs* Control; #*P* < 0.05, ##*P* < 0.01, ###*P* < 0.001 *vs* RANKL; Δ*P* < 0.05, ΔΔ*P* < 0.01, ΔΔΔ*P* < 0.001 *vs* RANKL + LPS (*n* = 3).

Next, we investigated the role of MCC950 in modulating LPS-induced inflammatory responses and autophagy in RAW264.7 cells. Quantitative PCR and immunoblotting analyses revealed that, compared with the RANKL-only control, the RANKL + LPS group exhibited markedly elevated mRNA levels of the inflammatory genes IL-1β and NLRP3, as well as increased NLRP3 protein expression, indicating that LPS triggers inflammatory activation (Fig. 1B; Fig. 2A, B). Although the differences did not reach statistical significance, the RANKL + LPS + MCC950 group showed reduced IL-1β and NLRP3 transcript levels relative to the RANKL + LPS group (Fig. 1B). In contrast to the RANKL control, treatment with RANKL + LPS significantly upregulated the expression of the autophagy-related genes and proteins LC3B and p62. Conversely, the RANKL + LPS + MCC950 combination not only suppressed LC3B and p62 gene expression but also decreased p62 protein levels, confirming that MCC950 attenuates autophagy during osteoclast differentiation under inflammatory conditions (Fig. 1C; Fig. 2A, B).

### 3.3 MCC950 inhibits osteoclast differentiation of RAW264.7 cells

To further assess the inhibitory effect of MCC950 on osteoclast differentiation induced by RANKL and LPS in RAW264.7 cells, we examined the expression of osteoclast-specific markers and performed TRAP staining. Quantitative PCR and immunoblotting analyses revealed that, relative to the control group, both RANKL alone and RANKL combined with LPS upregulated the expression of osteoclast-related genes (TRAF6, c-Jun, and NFATc1) as well as c-Jun protein. Notably, RANKL + LPS treatment led to more pronounced increases in TRAF6, c-Jun, and NFATc1 transcript levels and c-Jun protein abundance than RANKL treatment alone. In contrast, addition of MCC950 to the RANKL + LPS combination (RANKL + LPS + MCC950) significantly suppressed the expression of these genes and proteins compared with the RANKL + LPS group (Fig. 1D; Fig. 2A, B).

TRAP staining revealed that RAW264.7 cells in the RANKL and RANKL + LPS groups underwent fusion, resulting in larger, irregularly shaped cells characterized by numerous reddish-purple granules in the cytoplasm and an increased nuclear count. In contrast, the RANKL + MCC950 and RANKL + LPS + MCC950 groups showed significant inhibition of osteoclast differentiation, with a marked reduction in the number of TRAP-positive cells. These findings indicate that MCC950 effectively inhibits RANKL and RANKL + LPS-induced osteoclast differentiation in RAW264.7 cells (Fig. 2C and D).

### 3.4 High-dose LPS suppresses viability of MSCs (C3H10)

**Fig. 3:**
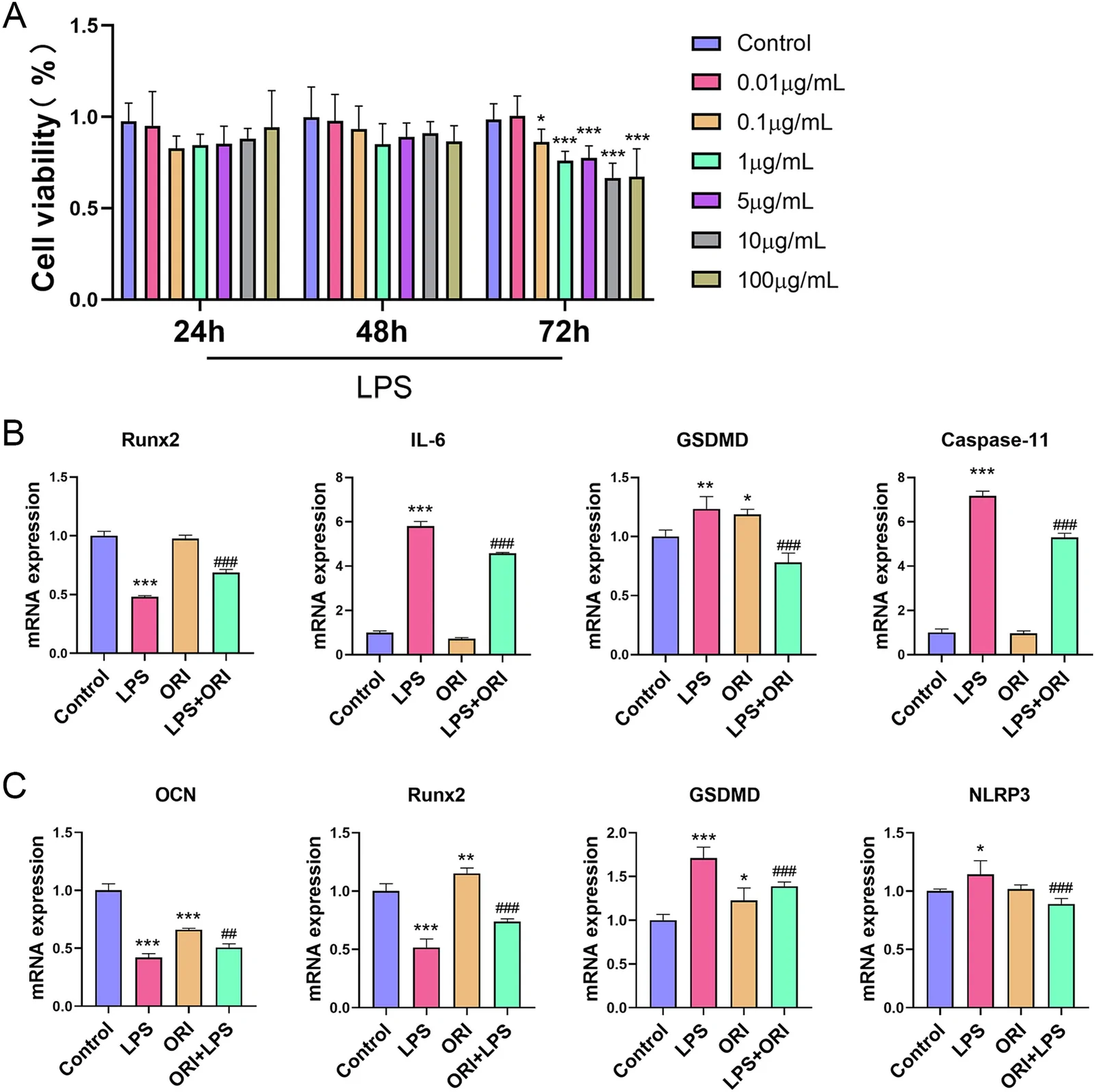
Effects of LPS and ORI treatment on MSCs (C3H10). (**A**) Cell viability of MSCs measured by CCK-8 assay after LPS treatment. (**B**) Gene expression of *Runx2, IL-6, GSDMD,* and *Caspase-11* in MSCs treated with LPS and ORI for 24 h. (**C**) Gene expression of *OCN, Runx2, GSDMD,* and *NLRP3* in MSCs treated with LPS and ORI for 7 d. *P < 0.05, **P < 0.01, ***P < 0.001 *vs* Control; #*P* < 0.05, ##*P* < 0.01, ###*P* < 0.001 *vs* ORI; Δ*P* < 0.05, ΔΔ*P* < 0.01, ΔΔΔ*P* < 0.001 *vs* ORI + LPS (*n* = 3).

To establish a safe concentration range of LPS for use in MSCs (C3H10), we first evaluated its cytotoxic effects using the CCK-8 assay. As demonstrated, LPS suppresses cell viability in a concentration- and time-dependent manner until this saturation point is reached. At 72 h, 10 μg/mL LPS significantly reduced cell viability relative to the vehicle control (Fig. 3A). Therefore, to minimize unwanted cytotoxicity, a concentration of 10 μg/mL was chosen for subsequent induction of the inflammatory model in MSCs.

### 3.5 Oridonin attenuates LPS-induced inflammation and pyroptosis in MSCs

**Fig. 4:**
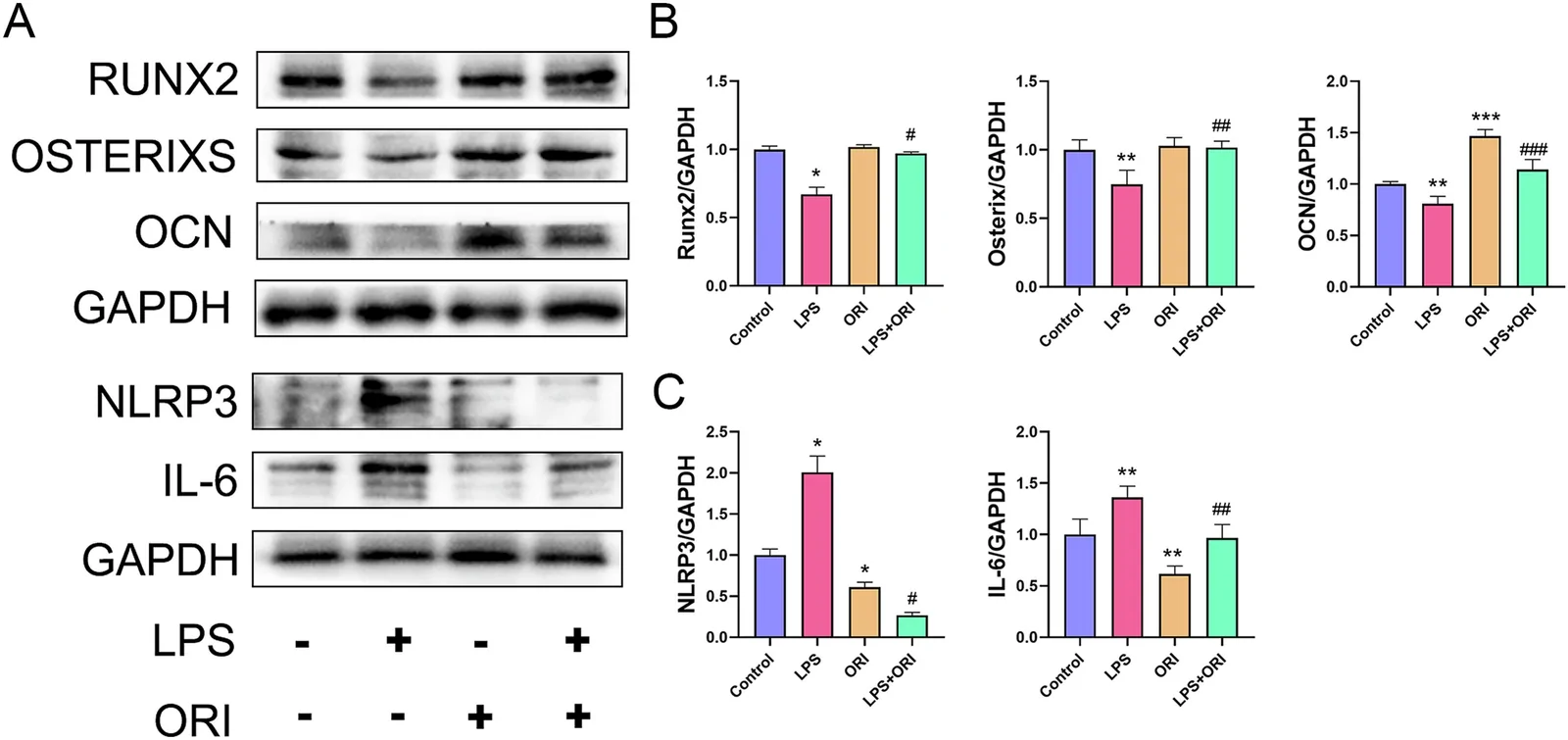
Oridonin promotes osteogenic differentiation of MSCs (C3H10). (**A**) Protein expression of Runx2, Osterix, OCN, NLRP3, and IL-6 in MSCs treated with LPS and ORI for 7 d. (**B**) The protein expression levels of Runx2, Osterix, and OCN in cells were quantitatively analyzed.(C) The protein expression levels of NLRP3 and IL-6 in cells were quantitatively analyzed.*P < 0.05, **P < 0.01, ***P < 0.001 *vs* Control; #*P* < 0.05, ##*P* < 0.01, ###*P* < 0.001 *vs* LPS (*n* = 3).

Next, we examined the role of ORI in mitigating LPS-induced inflammatory responses and pyroptosis in C3H10 cells. Quantitative PCR analysis revealed that, relative to the Control group, the LPS-treated group exhibited significantly elevated mRNA levels of the inflammation-associated genes *IL-6* and *NLRP3*, as well as the pyroptosis-related genes *GSDMD* and *Caspase-11*. Notably, co-treatment with ORI (LPS + ORI group) led to a marked reduction in the expression of *IL-6, NLRP3*, *GSDMD*, and *Caspase-11* compared with the LPS group alone (Fig. 3B, C). Immunoblotting further demonstrated that LPS upregulated the protein levels of the inflammasome component NLRP3 and the pro-inflammatory cytokine IL-6 relative to the Control group. Conversely, these increases were substantially attenuated in the LPS + ORI group when compared to the LPS-only condition (Fig. 4A, C).

### 3.6 Oridonin promotes osteogenic differentiation of MSCs (C3H10)

To further explore how ORI counteracts the suppressive effect of LPS on osteogenic differentiation of MSCs(C3H10), we assessed the expression of key osteogenesis-related markers, including *Runx2* and *OCN,* at the mRNA level, as well as OCN, Osterix, and Runx2 at the protein level. Quantitative PCR analysis showed that LPS-induced inflammation significantly reduced the transcript levels of *Runx2* and *OCN*. In contrast, co-treatment with ORI (LPS + ORI group) markedly restored the expression of these genes (Fig. 3B, C). Immunoblotting further revealed that LPS stimulation substantially suppressed the protein levels of OCN, Osterix, and Runx2, whereas ORI treatment significantly promoted their expression. Collectively, these findings indicate that ORI effectively alleviates the LPS-mediated impairment of osteogenic differentiation in MSCs (Fig. 4A, B).

### 3.7 Effect of MCC950 on femoral biomechanical properties in mice

**Fig. 5:**
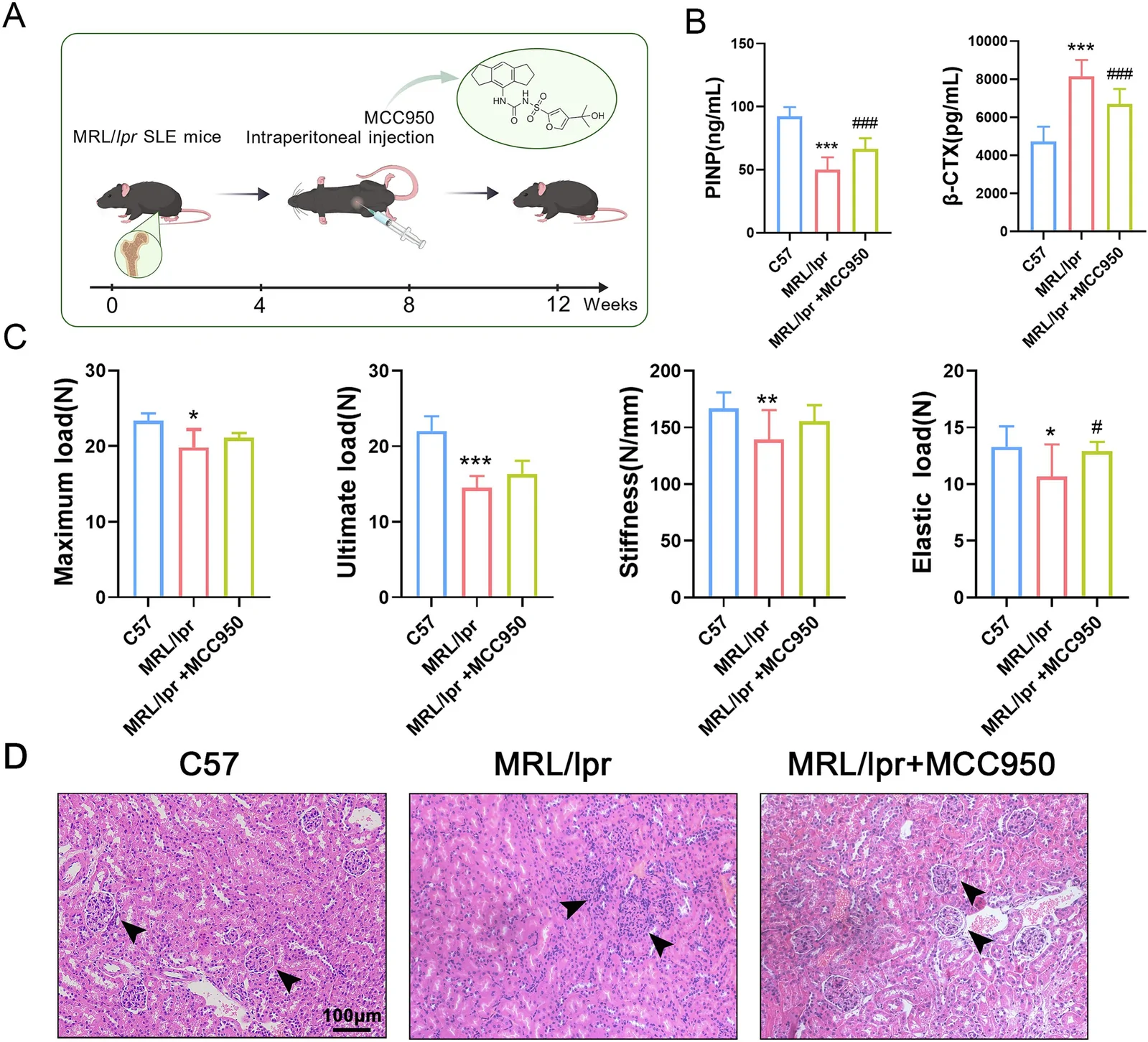
MCC950 ameliorates bone loss and glomerular inflammation in MRL/*lpr* mice. (**A**) Schematic illustration of the study design. (**B**) Effects of MCC950 on serum levels of PINP and β-CTX in mice. (**C**) Effects of MCC950 on maximum load, ultimate load, stiffness, and elastic load of the mouse femur. (D) Effect of MCC950 on mouse glomerular histology (H&E staining).*P < 0.05, **P < 0.01, ***P < 0.001 *vs* C57; #*P* < 0.05, ##*P* < 0.01, ###*P* < 0.001 *vs* MRL/*lpr* (*n* = 10).

The evaluation of the mechanical properties of femoral samples indicated that stiffness, elastic load, maximum load, and ultimate load were significantly lower in the MRL*/lpr* group than in the C57 group. In contrast, the MRL*/lpr* + MCC950 group exhibited a significantly higher elastic load than the MRL*/lpr* group (*P* < 0.05), whereas stiffness, maximum load, and ultimate load demonstrated non-significant increasing trends. These results suggest a reduction in bending resistance and an increase in bone brittleness in MRL*/lpr* mice, which were improved following MCC950 treatment (Fig. 5C).

### 3.8 Effect of MCC950 on femoral microstructure in mice

**Fig. 6:**
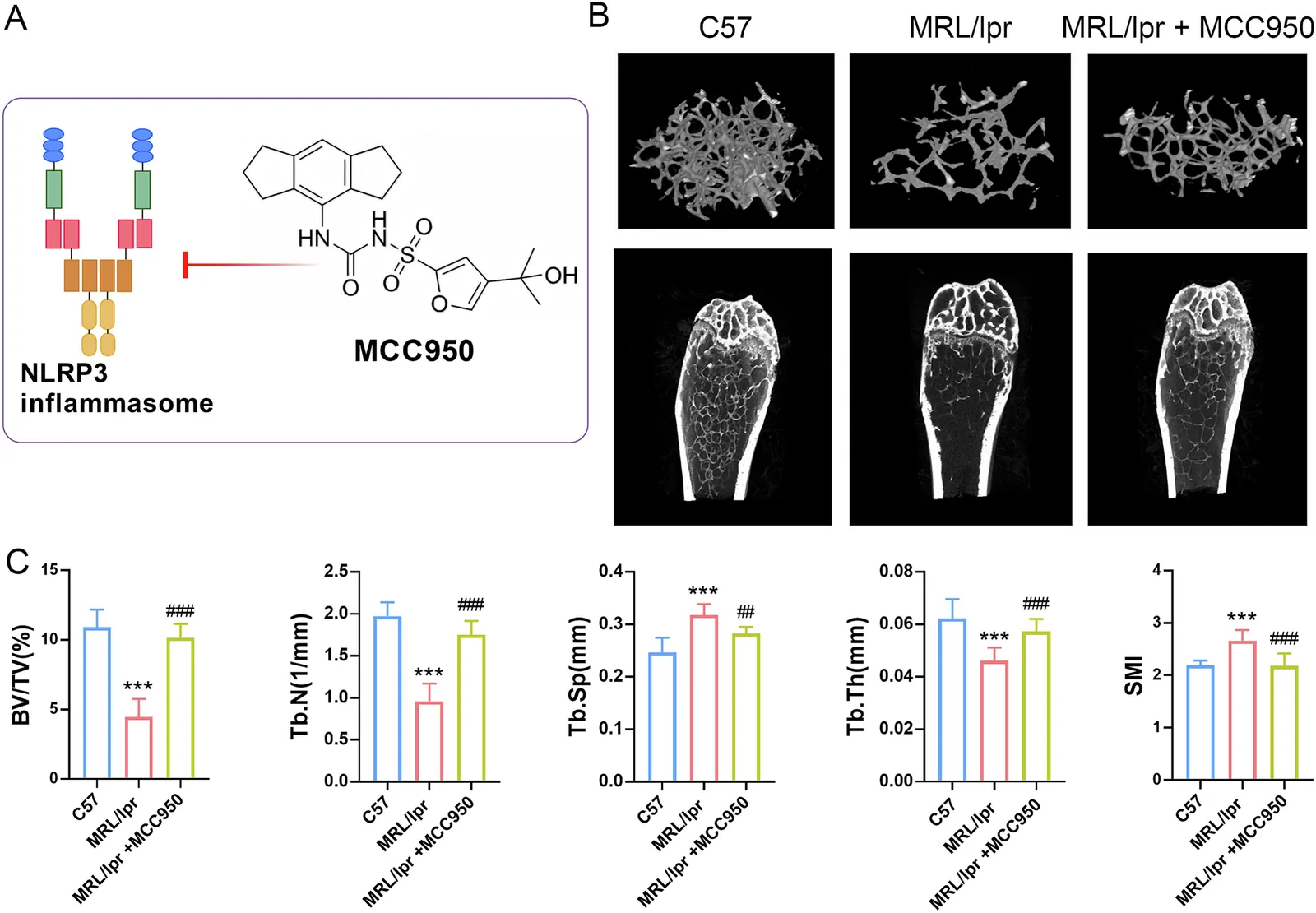
MCC950 ameliorated inflammation-induced bone loss *in vivo*. (**A**) MCC950 inhibits osteoclast differentiation by suppressing NLRP3 activation. (**B**) Representative 3D Micro-CT reconstructions of trabecular bone in the mouse femur. (**C**) Microstructural parameters:BV/TV, Tb.Th, Tb.N, Tb.Sp, SMI. *P < 0.05, **P < 0.01, ***P < 0.001 *vs* C57; #*P* < 0.05, ##*P* < 0.01, ###*P* < 0.001 *vs* MRL/*lpr* (*n* = 10).

Micro-CT analysis revealed that, relative to the C57 control group, the MRL*/lpr* group exhibited significantly lower BV/TV, Tb.Th), and Tb.N, along with a marked increase in Tb.Sp and SMI. In contrast, treatment with MCC950 significantly elevated BV/TV, Tb.Th, and Tb.N while reducing Tb.Sp and SMI compared with the MRL*/lpr* group. These findings confirm the development of osteoporotic changes in MRL*/lpr* mice and demonstrate that MCC950 effectively ameliorates this osteoporotic phenotype (Fig. 6B, C). Moreover, these micro-CT results were broadly consistent with the observed biomechanical property measurements.

### 3.9 Effects of MCC950 on serum osteoporosis biomarkers in mice

Serum biochemical analysis revealed that, relative to the C57 control group, the MRL*/lpr* group had significantly lower circulating levels of PINP and elevated levels of β-CTX, indicating increased bone resorption and reduced bone formation. In contrast, treatment with MCC950 (MRL*/lpr* + MCC950 group) led to increased PINP and decreased β-CTX levels compared with the untreated MRL*/lpr* group, suggesting that MCC950 promotes bone formation while suppressing bone resorption. Collectively, these findings demonstrate that the changes in bone metabolism markers in MRL*/lpr* mice are consistent with the osteoporotic phenotype observed by micro-CT analysis, and that MCC950 treatment effectively reverses these abnormalities (Fig. 5B).

### 3.10 Effect of oridonin on femoral biomechanical properties in mice

**Fig. 7:**
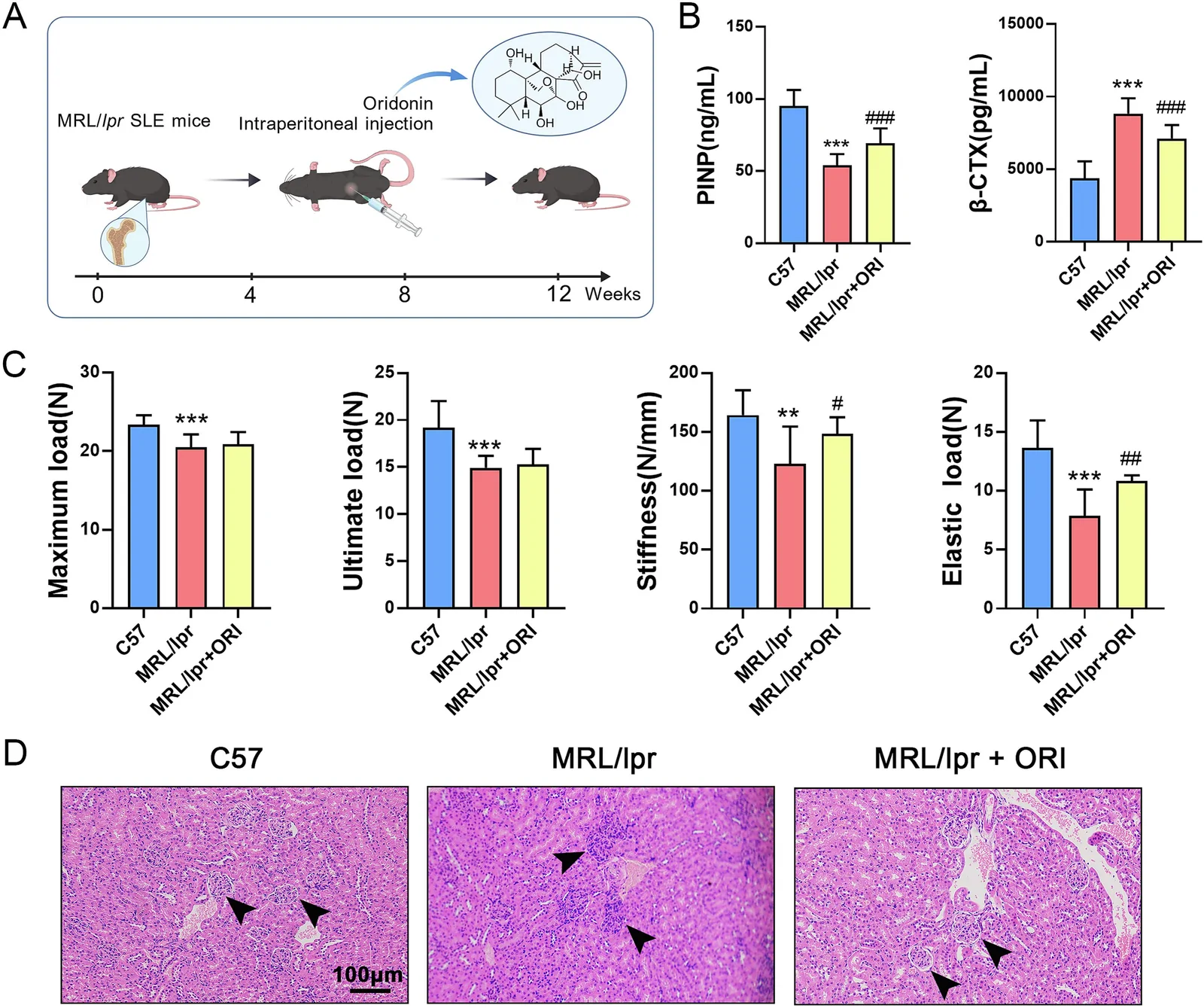
Oridonin ameliorates bone loss and glomerular inflammation in MRL/*lpr* mice. (**A**) Schematic illustration of the study design. (**B**) Effects of oridonin on serum levels of PINP and β-CTX in mice. (**C**) Effects of oridonin on maximum load, ultimate load, stiffness, and elastic load of the mouse femur. (**D**) Effect of oridonin on mouse glomerular histology (H&E staining). *P < 0.05, **P < 0.01, ***P < 0.001 *vs* C57; #*P* < 0.05, ##*P* < 0.01, ###*P* < 0.001 *vs* MRL/*lpr* (*n* = 10).

Biomechanical testing revealed that the MRL*/lpr* group displayed significantly diminished biomechanical properties of the femur compared to the C57 group, including maximum load, ultimate load, stiffness, and elastic load. Treatment with ORI notably enhanced stiffness and elastic load in MRL*/lpr* mice, and non-significant upward trends were also observed in the maximum and ultimate loads. These findings indicate that ORI effectively improves femoral stiffness and compressive strength in MRL*/lpr* mice, thereby enhancing the overall biomechanical properties of the femur (Fig. 7C).

### 3.11 Effect of oridonin on femoral microstructure in mice

**Fig. 8:**
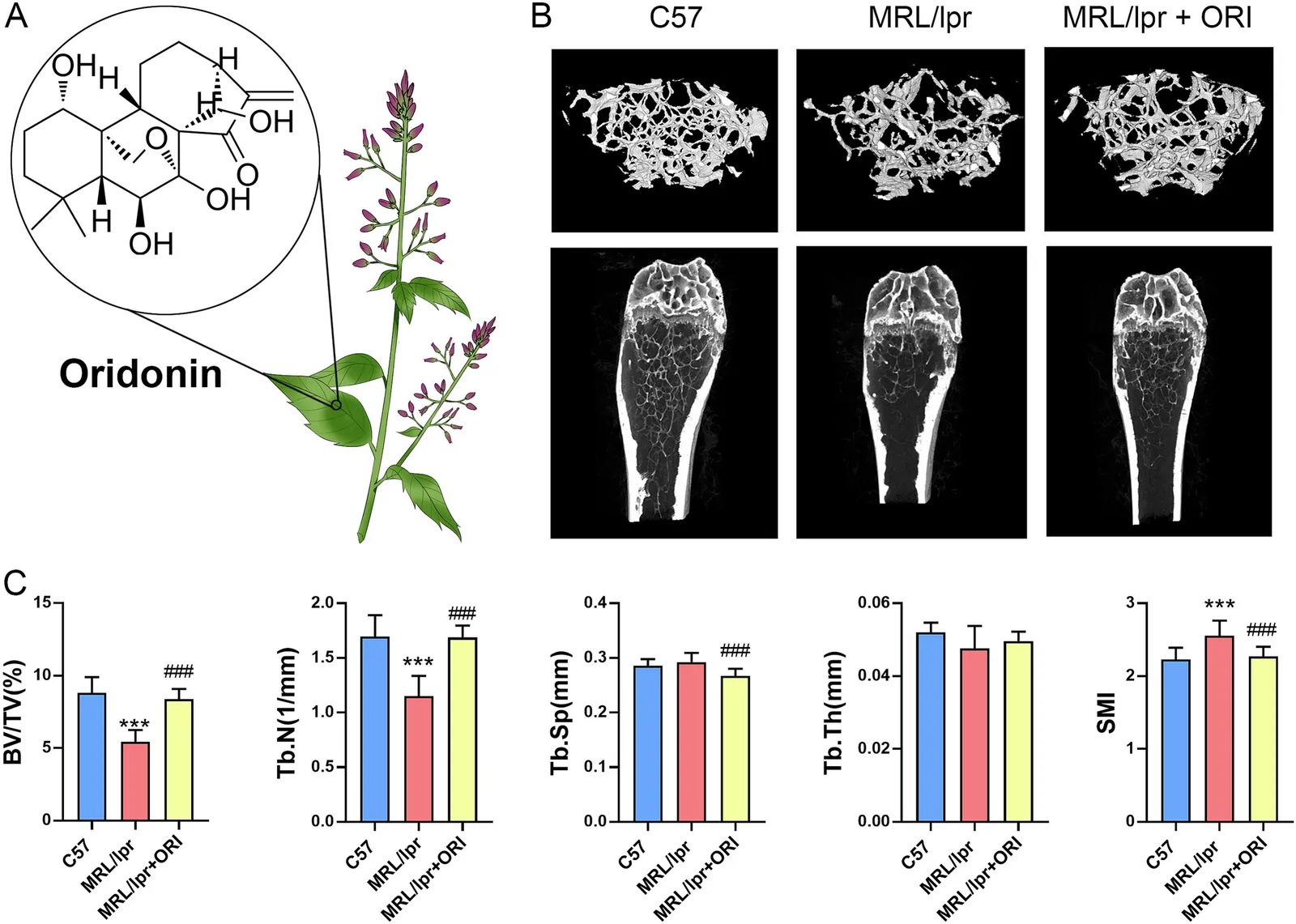
Oridonin ameliorated inflammation-induced bone loss *in vivo*. (**A**) Oridonin isolated from Isodon rubescens (Hemsl.) H.Hara. (**B**) Representative 3D Micro-CT reconstructions of trabecular bone in the mouse femur. (**C**) Microstructural parameters: BV/TV, Tb.Th, Tb.N, Tb.Sp, SMI. *P < 0.05, **P < 0.01, ***P < 0.001 *vs* C57; #*P* < 0.05, ##*P* < 0.01, ###*P* < 0.001 *vs* MRL/*lpr* (*n* = 10).

Micro-CT analysis revealed that the MRL*/lpr* group exhibited significantly lower BV/TV and Tb.N, and a significantly higher SMI than the C57 group. In contrast, the MRL*/lpr* + ORI group demonstrated significant increases in BV/TV and Tb.N, along with significant decreases in SMI and Tb.Sp compared to the MRL*/lpr* group. These findings indicate that, relative to the C57 group, the MRL*/lpr* group experienced increased trabecular separation, reduced trabecular number, and heightened trabecular fragmentation, reflecting severe disruption of the bone microarchitecture. Treatment with ORI in the MRL*/lpr* + ORI group markedly improved trabecular microstructure, approaching levels comparable to those of the C57 group, collectively suggesting that ORI effectively ameliorates osteoporosis in MRL*/lpr* mice (Fig. 8B, C).

### 3.12 Effects of oridonin on serum osteoporosis biomarkers in mice

Serum biochemical analysis revealed that MRL*/lpr* mice had lower serum PINP level and higher β-CTX level than C57 mice. After 12 weeks of ORI treatment, MRL*/lpr* mice exhibited elevated PINP and decreased β-CTX levels. These findings indicate that ORI enhances bone formation while inhibiting bone resorption in MRL*/lpr* mice (Fig. 7B).

### 3.13 MCC950 and oridonin ameliorate glomerular inflammation and injury in MRL/*lpr* mice

Histological examination of H&E-stained kidney sections revealed distinct glomerular alterations in the SLE model group. In C57 control mice, the glomeruli displayed normal cellularity and well-defined architecture (black arrows). In contrast, the MRL/lpr group exhibited pronounced glomerular damage, characterized by marked inflammatory cell infiltration, mesangial expansion, and loss of structural integrity (black arrows). Notably, treatment with either MCC950 or ORI substantially attenuated these pathological changes, with most glomeruli showing restored morphology and reduced inflammatory infiltrates, comparable to those observed in the healthy controls (black arrows). These findings collectively indicate that both NLRP3 inhibitors effectively mitigate the glomerular injury associated with the lupus-prone phenotype in MRL/lpr mice (Fig. 5D, 7D).

## 4 Discussion

Systemic inflammation in SLE can lead to bone loss by increasing osteoclast-mediated bone resorption and decreasing osteogenesis (Liu et al., 2016). Evidence indicates that the NLRP3 inflammasome plays a critical role in the pathogenesis and progression of SLE. Several studies involving both humans and various animal models have demonstrated hyperactivation of the NLRP3 inflammasome in SLE (Li et al., 2020; Tan et al., 2019; Ren et al., 2024). Overactivated NLRP3 inflammasome not only promote bone resorption but also suppress bone formation (Jiang et al., 2021). However, whether NLRP3 inhibition can ameliorate osteoporosis in SLE has not been confirmed previously. In this study, we employed two NLRP3 inhibitors, a synthesized commercial NLRP3 inhibitor (MCC950) and a natural NLRP3 inhibitor (ORI), to demonstrate that suppression of NLRP3 effectively alleviates SLE-associated glomerular inflammation and bone loss in MRL/lpr mice. Prior evidence demonstrates that MCC950 does not influence osteoblast-specific gene expression during differentiation (Cai et al., 2024). To maximize experimental relevance, we therefore exclusively evaluated MCC950 in RAW cells to isolate its effects on osteoclastogenesis within an inflammatory microenvironment. Conversely, the capacity of Oridonin to inhibit inflammation-induced osteoclast differentiation—primarily *via* DC-STAMP suppression—has been well characterized in existing literature (Zou et al., 2021).To build upon this and avoid redundant findings, our study strategically focused on the less-explored impact of Oridonin on the osteogenic potential of C3H10 mesenchymal stem cells under inflammatory stress.

MCC950 is a synthetic, highly specific small-molecule inhibitor of the NLRP3 inflammasome. Mechanistically, it directly binds the NACHT domain of NLRP3, obstructing the NLRP3–NEK7 interaction and suppressing downstream ASC oligomerization without affecting other inflammasomes such as NLRC4 or AIM2 (Coll et al., 2015; Coll et al., 2019; Li et al., 2022b). Previous studies have demonstrated that MCC950 effectively inhibits NLRP3 inflammasome-mediated caspase-1 activation and IL-1β production and attenuates age-dependent alveolar bone loss by suppressing osteoclastogenesis (Ni et al., 2021). Our results similarly confirmed that MCC950 inhibits the NLRP3 inflammasome protein and RANKL expression. Furthermore, we investigated the mRNA expression of TRAF6, c-JUN, NFATc1 within the RANKL signaling pathway (Grigoriadis et al., 1994; Walsh et al., 2015; Park et al., 2017). We found that inflammation promotes the expression of osteoclast-related genes, whereas MCC950 significantly reduces their expression and suppresses inflammation. Notably, the expression of osteoclast-related genes was lower in the RANKL+MCC950 group than in the RANKL+LPS + MCC950 group, indicating that MCC950 can suppress osteoclast-related gene expression even in the absence of high inflammatory conditions. Consistent with the PCR results, western blot analysis of c-JUN protein revealed similar results. TRAP staining further validated these findings. These results collectively demonstrate that MCC950 inhibits osteoclast differentiation beyond suppression of inflammation.

Under physiological conditions, low-level autophagy maintains osteoclast homeostasis. However, excessive autophagy induced by hypoxia, inflammation, glucocorticoids, and estrogen deficiency promotes osteoclast activation and differentiation (Montaseri et al., 2020; Zhao et al., 2023). Numerous studies have demonstrated an interplay between the NLRP3 inflammasome and autophagy: appropriate autophagy can inhibit NLRP3 inflammasome activation, whereas NLRP3 inflammasome activation can regulate autophagy induction (Biasizzo and Kopitar-Jerala, 2020). Zoledronic acid has been reported to inhibit lipopolysaccharide (LPS)-induced osteoclastogenesis by suppressing the NLRP3-mediated autophagy pathway (Cheng et al., 2024). The p62 protein is recognized as an autophagosome marker. Our study found that the p62 protein level was decreased in the RANKL+LPS + MCC950 group compared to that in the RANKL+LPS group. This suggests that MCC950 not only inhibits inflammation but also indirectly suppresses osteoclast activation and function by inhibiting osteoclast autophagy. Interestingly, in our study, we observed that the LC3-II/LC3-I protein ratio was significantly lower in the RANKL+LPS group compared to the RANKL+LPS + MCC950 group in Western blot analysis, a result contrasting with the qPCR data. This may be attributed to the sequestration of excess LC3-I by p62 aggregates in autophagy-impaired cells, preventing its conversion to LC3-II (Runwal et al., 2019). In our study, MCC950 exerted anti-resorptive effects primarily by inhibiting NLRP3-dependent autophagy and osteoclast differentiation in RAW264.7 cells. This observation is consistent with that of Cai et al., who reported that MCC950 suppresses RANKL-induced osteoclastogenesis in bone marrow monocytes (BMMs) *via* the NF-κB/c-Fos/NFATc1 signaling pathway. The study further noted that MCC950 does not significantly alter the osteogenic differentiation of bone marrow mesenchymal stem cells (BMSCs) under baseline physiological conditions (Cai et al., 2024), highlighting its lineage-selective, osteoclast- predominant action in homeostatic bone remodeling. Although MCC950 exhibits minimal direct pro-osteogenic activity under non-inflammatory conditions, Wang et al. demonstrated that under pathological stress (such as cigarette smoke extract (CSE)-induced damage), MCC950 effectively rescues impaired BMSC osteogenesis and mineralization by blocking NLRP3-mediated pyroptosis (Wang et al., 2024). This indicates that MCC950 preserves osteoblastic function predominantly when the NLRP3/pyroptotic pathway is aberrantly hyperactivated.

Oridonin (ORI) is a specific nature inhibitor of NLRP3 (Zhao et al., 2020), which inhibits NLRP3 by covalently binding to the Cys279 residue within its NACHT domain. In contrast to the targeted profile of MCC950, oridonin is a natural ent-kaurene diterpenoid with inherently broader polypharmacological activity. Beyond NLRP3 inhibition, oridonin directly engages multiple signaling cascades, including Wnt/β-catenin activation and NF-κB/MAPK attenuation (Zou et al., 2021; Zhou et al., 2020). Oridonin can mitigate ovariectomy (OVX)-induced osteoporosis by inhibiting osteoclastogenesis (Xie et al., 2018). ORI can enhance the proliferation and osteogenic differentiation of LPS-induced human periodontal ligament stem cells (hPDLSCs) in an inflammatory environment, thereby alleviating periodontitis (Jiang et al., 2023). Originally, ORI was approved for chronic tonsillitis, pharyngitis, laryngitis, and stomatitis in China. Consequently, while its long-term safety profile is firmly established in clinical practice, its therapeutic utility in SLE, particularly regarding SLE-driven bone loss, has yet to be realized. There was no comprehensive study covering this point. In this study, we investigated the effect of ORI on mesenchymal stem cells (C3H10 cells) in an inflammatory environment. In the present study, compared with the LPS-only group, cells co-treated with ORI (LPS + ORI) exhibited markedly elevated protein levels of Runx2, Osterix, and OCN, alongside a pronounced reduction in NLRP3 and IL-6 expression. These findings confirm that ORI effectively rescues osteogenic differentiation of mesenchymal stem cells (MSCs) from inflammatory suppression—a result that aligns with previous observations and highlights its therapeutic translatability for SLE. Furthermore, our data obtained from C3H10T1/2 cells, together with earlier evidence from Zhou et al., indicate that Oridonin concurrently attenuates osteoclastogenesis and promotes osteoblast differentiation by suppressing NLRP3 activation, modulating oxidative stress, and mitigating cellular apoptosis (Zhou et al., 2020).

Studies have shown that pyroptosis is closely linked to osteogenesis and bone loss (Li et al., 2023). Necrosulfonamide can reverse the inhibitory effects of pyroptosis on osteoblast proliferation and differentiation *via* the NLRP3/caspase-1/GSDMD pathway (Zhang and Wei, 2021). Our study found that ORI reduced the expression of *GSDMD*, *caspase-11*, and *NLRP3* genes, further confirmed by the evaluation NLRP3 and IL-6 protein levels, suggesting ORI inhibited inflammation, and the results of attenuated MSC pyroptosis may be related to the suppression of NLRP3 inflammasome.

Although the present study primarily focused on the direct effects of oridonin and MCC950 on osteoblastic (C3H10) and osteoclastic (RAW264.7) lineages, we acknowledge that the bone multicellular unit (BMU) represents a far more complex ecosystem. The therapeutic effects observed *in vivo* may also involve additional cell types, including osteocytes, osteal macrophages, and vascular endothelial cells (Kular et al., 2012).

Although direct investigations into Oridonin's effects on mature osteocytes remain limited, evidence indicates that Oridonin attenuates osteoblast apoptosis in osteoporotic rat models *via* the Hippo/YAP signaling pathway, pointing to a potential cytoprotective effect on osteocyte viability under metabolic stress (LU Haifeng, 2024). In inflammatory macrophage subsets, Oridonin suppresses NLRP3 inflammasome activation and reduces pro-inflammatory cytokine release in LPS-challenged BMMs (He et al., 2018). Because these macrophages share fundamental inflammasome signaling pathways with standard macrophages, Oridonin likely attenuates inflammatory macrophage subset-mediated inflammatory signaling, thereby indirectly preserving bone homeostasis. Oridonin exhibits context-dependent vascular dynamics. Under physiological or osteoporotic conditions, it facilitates angiogenesis by activating the Wnt3a/β-catenin/VEGF signaling pathway, improving microvascular perfusion, and supporting bone formation (Yu et al., 2023). Conversely, in pathological tumor models, Oridonin demonstrates anti-angiogenic capacity by disrupting VEGF-induced VEGFR2 signaling, thereby suppressing endothelial proliferation and tube formation (Zhou et al., 2021).

In human periodontal ligament cells (HPDLCs), MCC950 rescues LPS-impaired osteogenesis and restores the expression of key osteogenic markers, including ALP, Runx2, and OCN (Peng et al., 2021). In MLO-Y4 osteocytic cells, MCC950 mitigates BPA-induced pyroptosis by targeting the ROS/NLRP3/Caspase-1 axis (Zhang et al., 2022), demonstrating direct anti-pyroptotic protection in osteocytes. By specifically blocking the NLRP3 inflammasome in BMMs, MCC950 restricts the secretion of key inflammatory mediators, such as IL-1β. Within the bone microenvironment, this suppression attenuates macrophage-driven osteogenic inhibition while attenuating pro-osteoclastogenic signals. MCC950 also prevents NLRP3-mediated endothelial senescence and cellular dysfunction, suggesting a protective role in maintaining bone marrow microvascular integrity (Romero et al., 2022).

In our *in vitro* studies, LPS was employed as a standardized, well-validated reagent to trigger TLR4/NF-κB-dependent NLRP3 activation, allowing us to isolate the downstream cell-autonomous effects on osteogenesis and osteoclastogenesis, rather than serving as a pathogenic driver specific to systemic lupus erythematosus (SLE). In SLE-associated bone loss, chronic tissue damage is driven by a complex inflammatory milieu rich in endogenous danger signals and cytokines (*e.g.*, TNF-α, IL-6, and IL-1β).To corroborate the translational relevance of our *in vitro* observations, we subsequently validated the disease-specific pathological features *in vivo* using the well-established MRL/lpr autoimmune murine model. Recent studies have demonstrated that NLRP3 inhibitors effectively ameliorate SLE symptoms, including reducing proteinuria and renal inflammation (Chen et al., 2021). Lin Zhou et al. reported that ORI effectively ameliorated serological and clinical manifestations of SLE in MRL/*lpr* mice by inhibiting BAFF (Zhou et al., 2013). In this study, we investigated the potential of blocking NLRP3 for treating osteoporosis in MRL/*lpr* SLE mice. Our findings demonstrate that the NLRP3 inhibitors MCC950 and oridonin ameliorate glomerular inflammation and injury in MRL/lpr mice. Micro-CT and biomechanical analysis revealed that, compared to C57 mice, MRL/*lpr* mice exhibited decreased trabecular number and thickness, degenerated trabecular microstructure, reduced biomechanical properties, indicating the occurrence of osteoporosis and an elevated fracture risk. Additionally, serum biochemical assays further confirmed the disruption of bone metabolism in MRL/*lpr* mice. In MRL/lpr mice, NLRP3 inhibition (either MCC950 or oridonin) significantly improved trabecular bone microarchitecture, enhanced biomechanical performance, increased PINP levels, and decreased β-CTX levels. These findings collectively demonstrate that blocking NLRP3 confers therapeutic benefits against SLE-associated osteoporosis, revealing the efficacy of MCC950 and ORI in ameliorating SLE-associated osteoporosis.

## 5 Conclusion

In summary, our study demonstrates that inhibition of NLRP3 (either MCC950 or oridonin) ameliorated osteoporosis in SLE mice. We also found that ORI, as a natural NLRP3 inhibitor, it further promoted osteogenic differentiation of MSCs. These effects potentially through suppressing inflammation and pyroptosis of MSCs. The strategy of blocking NLRP3, especially using ORI (a commercial clinical medicine), hold promise as potential therapeutics for SLE-associated osteoporosis, may prone to a promising case of new use for old drug on clinical translation.

## CRediT authorship contribution statement

**Feifu Deng:** Writing – original draft, Methodology, Investigation, Formal analysis, Data curation, Project administration. **Yuting Liao:** Writing – original draft, Visualization, Validation, Software, Writing – review & editing. **Weixiong Guo:** Methodology, Investigation, Formal analysis, Data curation, Project administration. **Xiangxin Zhong:** Formal analysis, Data curation, Investigation, Methodology, Project administration. **Xiang Gao:** Funding acquisition, Formal analysis, Data curation, Writing – original draft. **Ahmad:** Writing – review & editing. **Yanzhi Liu:** Writing – review & editing, Supervision, Project administration, Funding acquisition, Conceptualization. **Jinsong Wei:** Writing – review & editing, Supervision, Conceptualization.

## Declaration of competing interest

The authors declare that they have no known competing financial interests or personal relationships that could have appeared to influence the work reported in this paper.

## Data Availability

Data will be made available on request.
