# Supplementary material for: Blockade of NLRP3 alleviates systemic lupus erythematosus-induced osteoporosis through dual modulation of osteogenesis and osteoclastogenesis
Source: Bone Rep. 2026 Aug 22;30:101942. doi: 10.1016/j.bonr.2026.101942 (PMC13543794; doi:10.1016/j.bonr.2026.101942)
Supplement: Supplementary file 1 [file mmc1.docx]

**Supplementary materials**

**Blockade of NLRP3 alleviates systemic lupus erythematosus-induced osteoporosis through dual modulation of osteogenesis and osteoclastogenesis**

Feifu Deng^a1^, Yuting Liao^b,c,d1^,Weixiong Guo^a1^, Xiangxin Zhong^a^, Xiang Gao^e^, Ahmad^b^, Yanzhi Liu^b,d*^, Jinsong Wei^a*^

^a^ Orthopedics department, The Affiliated Hospital of Guangdong Medical University, Zhanjiang, 524001, People’s Republic of China.

^b^ Key Laboratory of Traditional Chinese Medicine for the Prevention and Treatment of Infectious Diseases, Institute of Clinical Medicine, Zhanjiang Central Hospital, Guangdong Medical University (Central People’s Hospital of Zhanjiang), Zhanjiang, 524045, People’s Republic of China.

^c^ Marine Medical Research Institute of Zhanjiang, School of Ocean and Tropical Medicine, Guangdong Medical University, Zhanjiang, 524023, People’s Republic of China.

^d^ School of Pharmacy, Guangdong Medical University, Zhanjiang, 524023, People’s Republic of China.

^e^ Stem Cell Research and Cellular Therapy Center, The Affiliated Hospital of Guangdong Medical University, Zhanjiang, 524001, People’s Republic of China.

RT-PCR Primer sequence

| Name | Sequence ( 5′→ 3′) |
| --- | --- |
| *IL-6* | F: CTCCCAACAGACCTGTCTATAC  R: CCATTGCACAACTCTTTTCTCA |
| *NLRP3* | F: GCCGTCTACGTCTTCTTCCTTTCC  R: CATCCGCAGCCAGTGAACAGAG |
| *Caspase-11* | F: GGCTACGATGTGGTGGTGAAAGAG  R: ATGTGCTGTCTGATGTCTGGTGTTC |
| *GSDMD* | F: CGATGGGAACATTCAGGGCAGAG  R: ACACATTCATGGAGGCACTGGAAC |
| *Runx2* | F: CCTTCAAGGTTGTAGCCCTC  F: CCTTCAAGGTTGTAGCCCTC |
| *Osterix* | F:GACTACCCACCCTTCCCTCACTC  R:TAGACACTAGGCAGGCAGTCAGAC |
| *OCN* | F:CAAGCAGGAGGGCAATAAGGTAGTG  R:TGCGTTTGTAGGCGGTCTTCAAG |
| *NFATc1* | F: CATCCTTGCCTGCCCTTGACTG  R: TGAGCCCTGTGGTGAGACTTGG |
| *c-Jun* | F: AAGACCGTGTCAGGAGGCAGAG  R:CAGCCATCTTATTCCGTTCCCTTCG |
| *TRAF6* | F: GACTGCCCAACAGCTCCAATCC  R:AAGTGTCGTGCCAAGTGATTCCTC |
| *p62* | F: AGGAGGAGACGATGACTGGACAC  R: TTGGTCTGTAGGAGCCTGGTGAG |
| *LC3B* | F:CAAGCCTTCTTCCTCCTGGTGAATG  R: CCATTGCTGTCCCGAATGTCTCC |

Note：F: Forward Sequence; R: Reverse Sequence
